# ScLRTC: imputation for single-cell RNA-seq data via low-rank tensor completion

**DOI:** 10.1186/s12864-021-08101-3

**Published:** 2021-11-29

**Authors:** Xiutao Pan, Zhong Li, Shengwei Qin, Minzhe Yu, Hang Hu

**Affiliations:** Department of Mathematical Sciences, School of Science, Zhejiang Sci-Tech University, Hangzhou, 310018 China

**Keywords:** Single-cell RNA-seq, Data imputation, Low-rank tensor

## Abstract

**Background:**

With single-cell RNA sequencing (scRNA-seq) methods, gene expression patterns at the single-cell resolution can be revealed. But as impacted by current technical defects, dropout events in scRNA-seq lead to missing data and noise in the gene-cell expression matrix and adversely affect downstream analyses. Accordingly, the true gene expression level should be recovered before the downstream analysis is carried out.

**Results:**

In this paper, a novel low-rank tensor completion-based method, termed as scLRTC, is proposed to impute the dropout entries of a given scRNA-seq expression. It initially exploits the similarity of single cells to build a third-order low-rank tensor and employs the tensor decomposition to denoise the data. Subsequently, it reconstructs the cell expression by adopting the low-rank tensor completion algorithm, which can restore the gene-to-gene and cell-to-cell correlations. ScLRTC is compared with other state-of-the-art methods on simulated datasets and real scRNA-seq datasets with different data sizes. Specific to simulated datasets, scLRTC outperforms other methods in imputing the dropouts closest to the original expression values, which is assessed by both the sum of squared error (SSE) and Pearson correlation coefficient (PCC). In terms of real datasets, scLRTC achieves the most accurate cell classification results in spite of the choice of different clustering methods (e.g., SC3 or t-SNE followed by K-means), which is evaluated by using adjusted rand index (ARI) and normalized mutual information (NMI). Lastly, scLRTC is demonstrated to be also effective in cell visualization and in inferring cell lineage trajectories.

**Conclusions:**

a novel low-rank tensor completion-based method scLRTC gave imputation results better than the state-of-the-art tools. Source code of scLRTC can be accessed at https://github.com/jianghuaijie/scLRTC.

## Background

Over the past few years, with the explosive growth of scRNA sequence data, important biological discoveries have been progressively conducted. However, as impacted by the picogram level of RNAs in a single cell, RNA transcripts may be missed during the reverse transcription and amplification step, so the transcripts are not detected in the following sequencing, which is termed as the dropout problem [1]. The resulting gene-cell expression matrix will consist of numerous false zeros attributed to dropout events, which will corrupt the biological signal and impede downstream analyses (e.g., cell clustering, data visualization and cell trajectory inference). To reduce the impact of this problem, besides increasing the efficiency of transcription capture, an effective imputation algorithm for scRNA-seq data should be developed to predict missing values attributed to dropout events [2].

Existing single-cell imputation methods have two main types: one complies with the deep learning method. For instance, DeepImpute [3] was designed to impute the scRNA sequence by applying a deep neural network (DNN) with a dropout layer and loss function to learn patterns in the data. DCA [4] established an auto-encoder to model the distribution of genes with a zero-inflated negative binomial prior, and then attempted to predict the mean, standard deviation and dropout probability of genes. ScIGANs [5] adopted the generative adversarial networks (GANs) to learn the dependence of nonlinear genes and genes from complex multi-cell type samples, and then trained the neural network model to generate real expression profiles of defined cell types. However, due to the influence of the training set and the existence of over-fitting problem, these methods may generate the false-positive results in differential expression analyses [6]. Another type for single-cell imputation methods complies with the statistical algorithm. For instance, SAVER [7] exploited information across genes in the identical cell type with a Bayesian approach to recover true expression levels; it also measured the uncertainty of recovered values. MAGIC [8] performed a soft clustering after building a Markov transition matrix, and then replaced a gene’s raw expression with its weighted mean expression in a cluster. However, MAGIC also imputes the gene expression values that are not affected by dropout. Therefore, it may introduce the new bias into the data and possibly eliminate the meaningful biological variations. ScImpute [9] initially estimated the probability of an entry to be dropout with the use of a mixture model, and then imputed the potential dropout entries of a cell by employing the information from the gene expression of consistent cells. DrImpute [10] presented a clustering-based method and implemented a consensus strategy which estimated a value with several cluster priors or distance matrices and then imputed the data by aggregation. CMF-Impute [11] drew upon the similarity of cells and genes to build a collaborative matrix factorization-based model for imputing the dropout entries of a given scRNA-seq expression. ALAR [12] provided a low-rank approximation of the expression matrix using singular vector decomposition (SVD). McImpute [13] used the nuclear norm minimization to realize a matrix completion algorithm for the scRNA data imputation. A study [14] suggested that taking advantage of the presence of low-rank submatrix can improve the imputation performance compared to the traditional low-rank matrix restore methods. For example, PBLR [15] considered the cell grouping information and performed a bounded low-rank completion method for each group. ScLRTD [16] introduced the tensor into the imputation of single-cell datasets, but it is mainly aimed at the completion with single-cell multi-omics sequencing data and the result in the scRNA dataset is not better than MAGIC, because this tensor based method did not fully take advantage of the correlation of single-cell data. Liu et al. [17] proposed a definition for the tensor trace norm that generalizes the established definition of matrix trace norm. Similar to the matrix based imputation, the tensor based imputation is formulated as a convex optimization problem and is solved by three algorithms SiLRTC, FaLRTC and HaLRTC. Experimental comparisons show that these methods are more accurate and robust than other heuristic approaches (Tucker, Parafac and SVD), which can propagate the data structure to fill large missing regions.

In this paper, a novel low-rank tensor completion based method (scLRTC) is proposed for the scRNA-seq data imputation. Since scRNA-seq data commonly involve single cells from different cell types and single cells with the identical type normally exhibit the similar expression pattern, the underlying true expression matrix is reasonably assumed to be able to be approximated by a low-rank matrix [1]. Based on such an assumption, the similar expression patterns of a single cell are adopted to build a third-order tensor, and then the single-cell gene expression is restored by approximating the tensor rank. This method is applied to nine scRNA-seq datasets and four simulation datasets, and it is compared with several state-of-the-art methods (SAVER [7], MAGIC [8], scImpute [9], DrImpute [10], CMF-Impute [11], PBLR [15], WEDGE [18] and scGNN [19]). As revealed from considerable data analyses, the proposed method is capable of achieving more accurate imputation results and improving the downstream analysis.

## Results

The proposed imputation method is employed for nine published scRNA-seq datasets (i.e., Pollen [20], Usoskin [21], Yan [22], Zeisel [23], Mouse [24], PBMC [25], Chen [26], Loh [27] and Petropoulos [28]) and four simulation datasets generated from Splatter package [29], and it is compared with some popular methods (e.g., SAVER [7], MAGIC [8], scImpute [9], DrImpute [10], CMF-Impute [11], PBLR [15], WEDGE [18] and scGNN [19]). The method is largely evaluated from five aspects with cell subpopulation clustering, dimensionality reduction visualization, data masking evaluation, correlation analysis and differential expression analysis, and cell trajectory inference. The parameter setting of the respective dataset is listed in Table 1, and the parameter setting of the simulation dataset is presented in Table 2.

**Table 1:** Parameter settings corresponding to respective datasets in our experiment

Dataset	K	P	*α*	*ρ*	epsilon
Pollen	10	10	[1 1e-1 2e-3]	1e-5	1e-2
Usoskin	8	8	[1 1e-2 1e-3]	1e-5	1e-2
Yan	5	5	[1 1e-2 2e-3]	1e-5	1e-2
Zeisel	20	20	[1 1e-2 1e-3]	1e-5	1e-2
Mouse	10	10	[1 1e-2 1e-3]	1e-5	1e-2
PBMC	10	10	[1 1e-2 1e-3]	1e-5	1e-3
Chen	10	10	[1 1e-2 1e-3]	1e-5	1e-3
Loh	11	11	[1 1e-2 2e-3]	2e-4	1e-2
Petropoulos	10	10	[1 1e-2 1e-3]	1e-5	1e-2
Simulation dataset	15	8	[1 1e-1 1e-3]	1e-5	1e-5

**Table 2:** Parameter settings of simulation datasets generated from Splatter

Parameter	Simulation dataset	Parameter	Simulation dataset
version	1.10.1	dropout.type	“group”
nGenes	1000	method	“groups”
nCells	500	de.prob	c(0.05, 0.08, 0.01)
group.prob	c(0.3, 0.3, 0.4)	de.facLoc	0.5
dropout.shape	c (ds, ds, ds), ds ∈ {−0.3,0, 0.05,0.25}	de.facScale	0.8
dropout.mid	Default	dropout. present	Null

### Evaluating imputation accuracy through cell clustering

In the relevant research on the scRNA-seq dataset, cell clustering refers to one of the critical contents. There are many clustering algorithms (e.g., K-means and SC3 [30]). Among the mentioned methods, SC3 is recognized as an accurate unsupervised single-cell clustering tool that does not explicitly address dropout events for the scRNA-seq data. Thus, the proposed method together with other popular scRNA-seq imputation methods was added into the preprocessing step of SC3. Then we used the cell clustering accuracy measured by adjusted rand index (ARI) [31] and normalized mutual information (NMI) [32] to evaluate their performance, namely, the consistency between the inferred cell cluster and the real cell cluster.

**Fig. 1:**
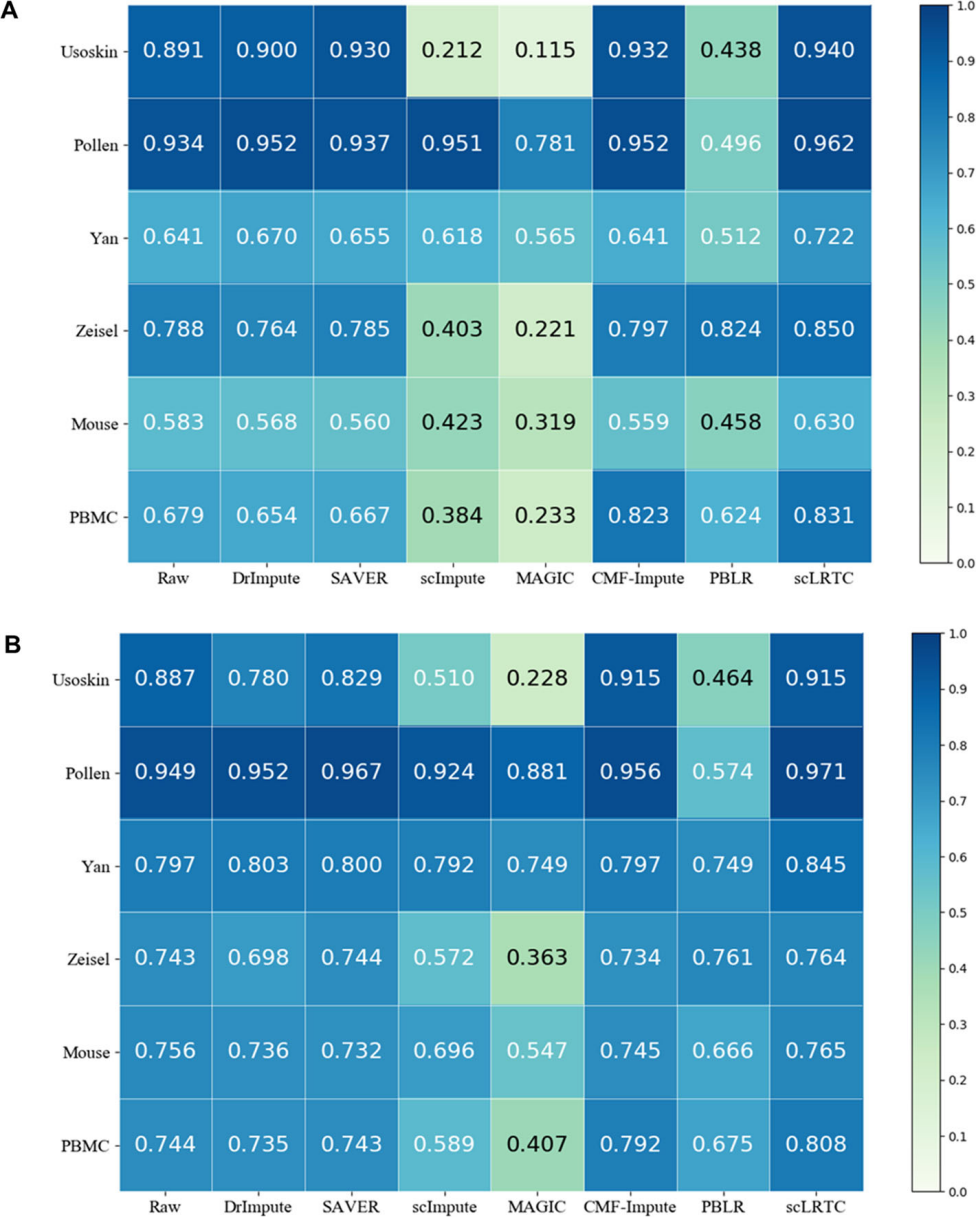
SC3 clustering comparison of scLRTC and other methods on different datasets. (**A**) Comparison of ARI indicators obtained by SC3 clustering on 6 scRNA-seq datasets using different algorithms. (**B**) Comparison of NMI indicators obtained by SC3 clustering on 6 scRNA-seq datasets using different algorithms

After the data imputation by the proposed scLRTC and other methods (DrImpute, SAVER, scImpute, MAGIC, CMF-Impute and PBLR), we used SC3 to cluster 6 published scRNA-seq datasets, including Usoskin, Pollen, Yan, Zeisel, Mouse and PBMC. The clustering accuracy measured by ARI and NMI are plotted in Fig. 1A and Fig. 1B, respectively. Obviously, the proposed method has the best ARI performance in Usoskin, Pollen, Yan, Zeiel, Mouse and PBMC, and the performance of NMI on the Usoskin dataset can be as competitive as CMF-Impute. In summary, the proposed method imputation can improve the clustering accuracy of SC3.

**Fig. 2:**
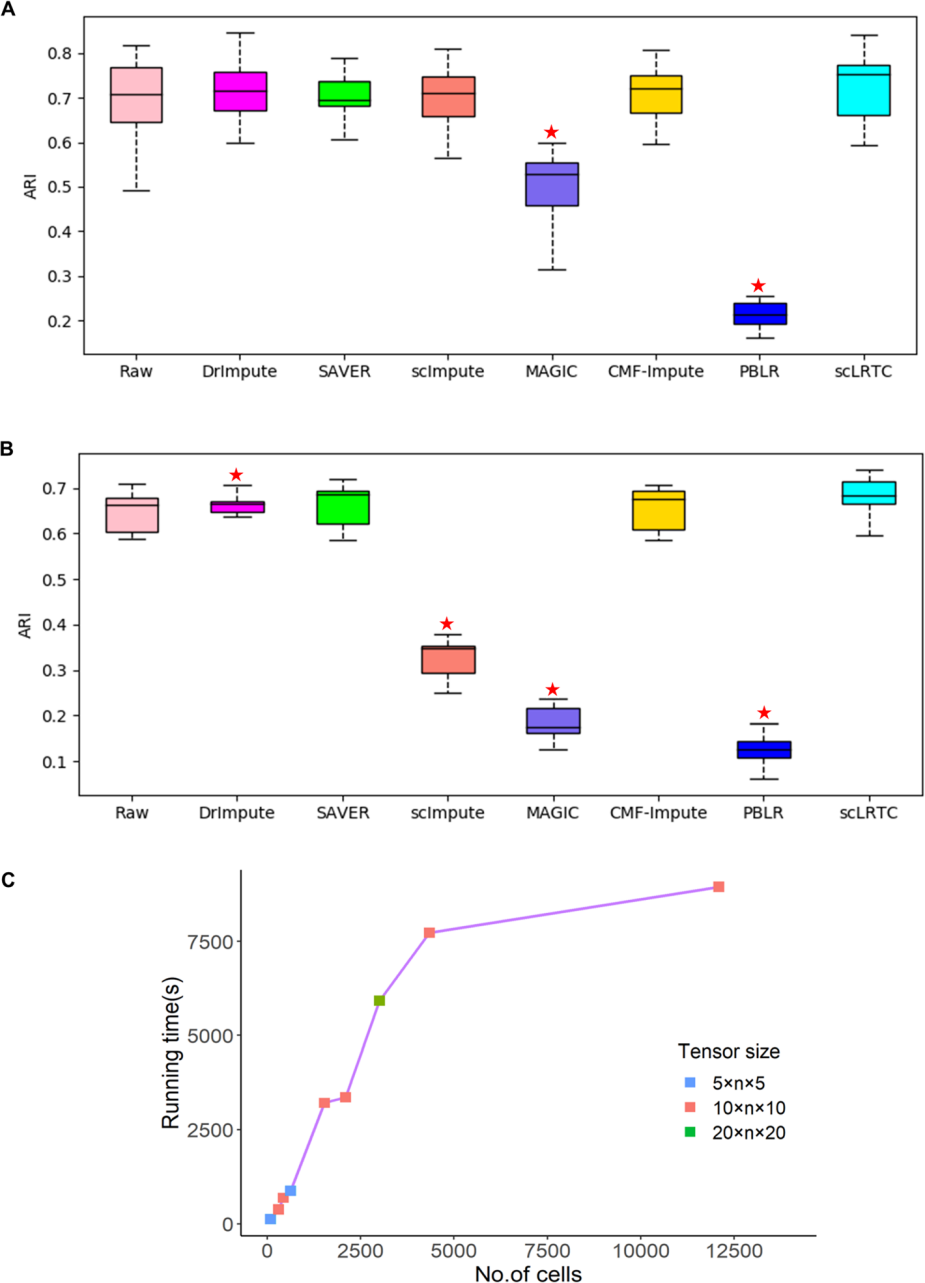
Performance analysis and comparison of scLRTC and other methods by the t-SNE + K-means clustering. (**A**) ARI obtained by t-SNE + K-means clustering on the Pollen dataset using different algorithms. (**B**) ARI obtained by t-SNE + K-means clustering on the Usoskin dataset using different algorithms. In (**A**) and (**B**), asterisk indicates the statistically significant difference (*P* < 0.05) between scLRTC and the imputation method of interest using the Wilcoxon rank-sum test. (**C**) Running time of scLRTC for datasets with different sample sizes. The different tensor size setting is represented by different colors, where *n* is the number of genes

To show that the proposed imputation method does not depend on the clustering method, we further used another popular single-cell clustering method (first using the dimensionality reduction by t-distributed stochastic neighbor embedding (t-SNE) [33], and then applying K-means for clustering) [10] to test the performance of the proposed scLRTC and other methods. Compared with the SC3 algorithm, the K-means algorithm is more affected by the initial values. To compare the clustering results more reasonably, we performed t-SNE + K-means 20 times on the Pollen and Usoskin datasets (The perplexity of t-SNE is set to 10, and other parameters of t-SNE are set as default parameters). In the Pollen dataset, our median is the highest at 0.722, and the maximum value is 0.853, which is better than the maximum value (0.847) by DrImpute (Fig. 2A). In the Usoskin dataset, the median of the proposed method is 0.684 and the maximum value is 0.742, which are both the highest compared to other methods (Fig. 2B). Note that the result of scImpute is worse than that of SAVER. When verifying the clustering performance by SC3 and t-SNE + K-means clustering, similar result was also appeared in CMF-Impute [11]. The main reason may be that scImpute relies on the spectral clustering which may influence the subsequent imputation process when the data has unbalanced clusters. In brief, the proposed imputation achieves a better overall effect than other imputation algorithms.

Furthermore, we compared scLRTC with the latest matrix completion based method WEDGE [18] and deep learning based method scGNN [19] on the Zeisel dataset. We applied the Scanpy’s Louvain algorithm [34, 35] for the scRNA-seq data clustering and found scLRTC achieved an ARI of 0.692, which is higher than WEDGE’s 0.560 and scGNN’s 0.678. Finally, we did the test for a large Chen dataset [26] where the number of cells is more than 10,000. We also used the Louvain algorithm to cluster the scRNA-seq data and found the clustering performance index ARI increased from 0.611 (raw data) to 0.673 by scLRTC. Considering the time complexity of scLRTC, the addition of tensor computation makes it slower than other methods. But we can control the size of tensor for various datasets to relief the influence of tensor computation. Figure 2C illustrates the running time of scLRTC for the mentioned experimental datasets with different sizes of tensor setting. It shows that the time complexity of scLRTC is not quadratic proportional to the number of cells, which makes it applicable for scRNA-seq datasets with different sizes.

### Cell visualization

**Fig. 3:**
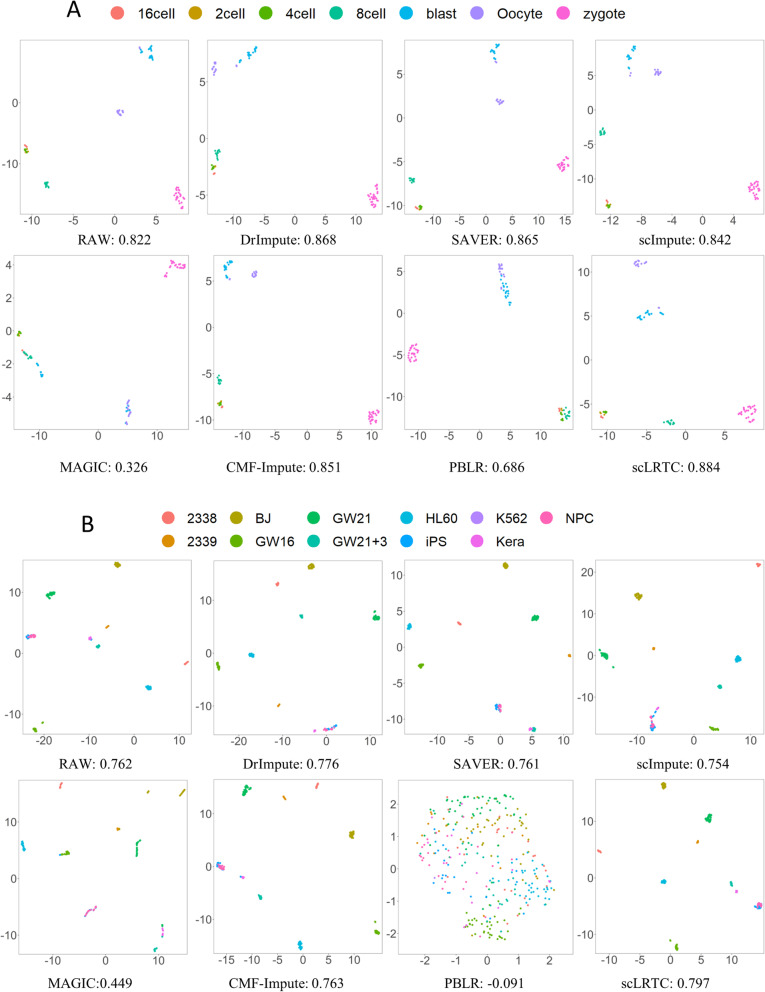
UMAP visualization and SC comparison on Yan and Pollen datasets. (**A**) UMAP visualization and SC of the Yan dataset. (**B**) UMAP visualization and SC of the Pollen dataset

**Fig. 4:**
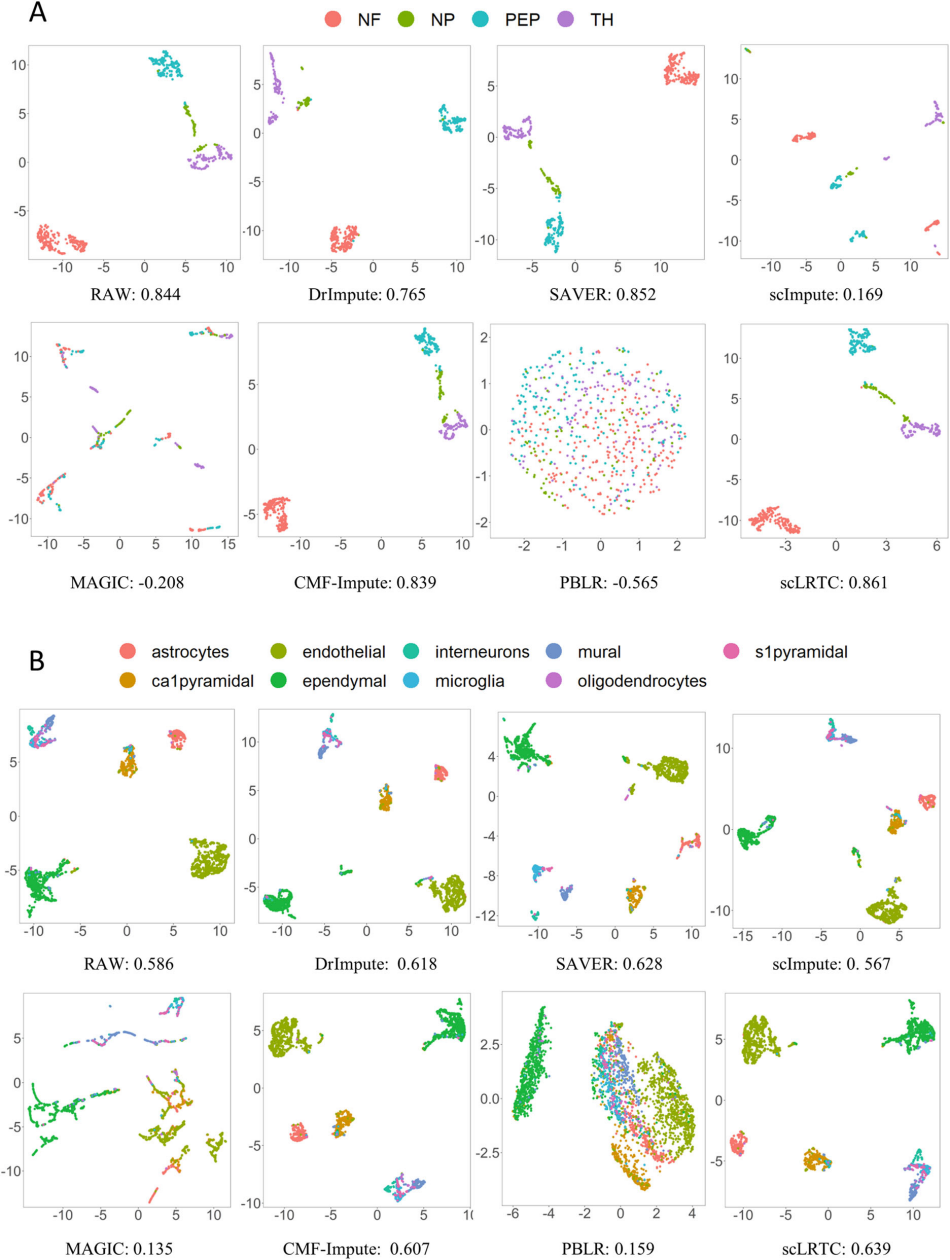
UMAP visualization and SC comparison on Usoskin and Zeisel datasets. (**A**) UMAP visualization and SC of the Usoskin dataset. (**B**) UMAP visualization and SC of the Zeisel dataset

Visually representing scRNA-seq data involves shrinking the gene expression matrix into a lower space, and then mapping each cell’s transcriptome in the reduced low dimensional space. Several dimensionality reduction methods are generally known (e.g., PCA [36], t-SNE and UMAP [37]), where UMAP is suggested to be particularly suitable for the visualization of any dimensional data. Accordingly, UMAP was employed to discuss the dimensionality reduction effect before and after imputation on four expression matrices of Yan, Pollen, Usoskin and Zeisel datasets. To be specific, cells were visualized in a two-dimensional space, and different cells were stained using real labels before and after imputation. To quantify the grouping of cell transcriptomes, an unsupervised clustering quality measurement was conducted with silhouette coefficient (SC) [38] to evaluate the effect of dimensionality reduction. The higher the silhouette coefficient, the more significant the dimensionality reduction effect will be. The UMAP dimensionality reduction visualization and the average SC of the raw and imputed data (4 published datasets) with different methods are illustrated in Fig. 3 and Fig. 4. According to these figures, the SC values of the proposed scLRTC in these datasets are the highest with 0.884, 0.797, 0.861, 0.639, respectively.

### Assessing imputation accuracy through data masking

**Fig. 5:**
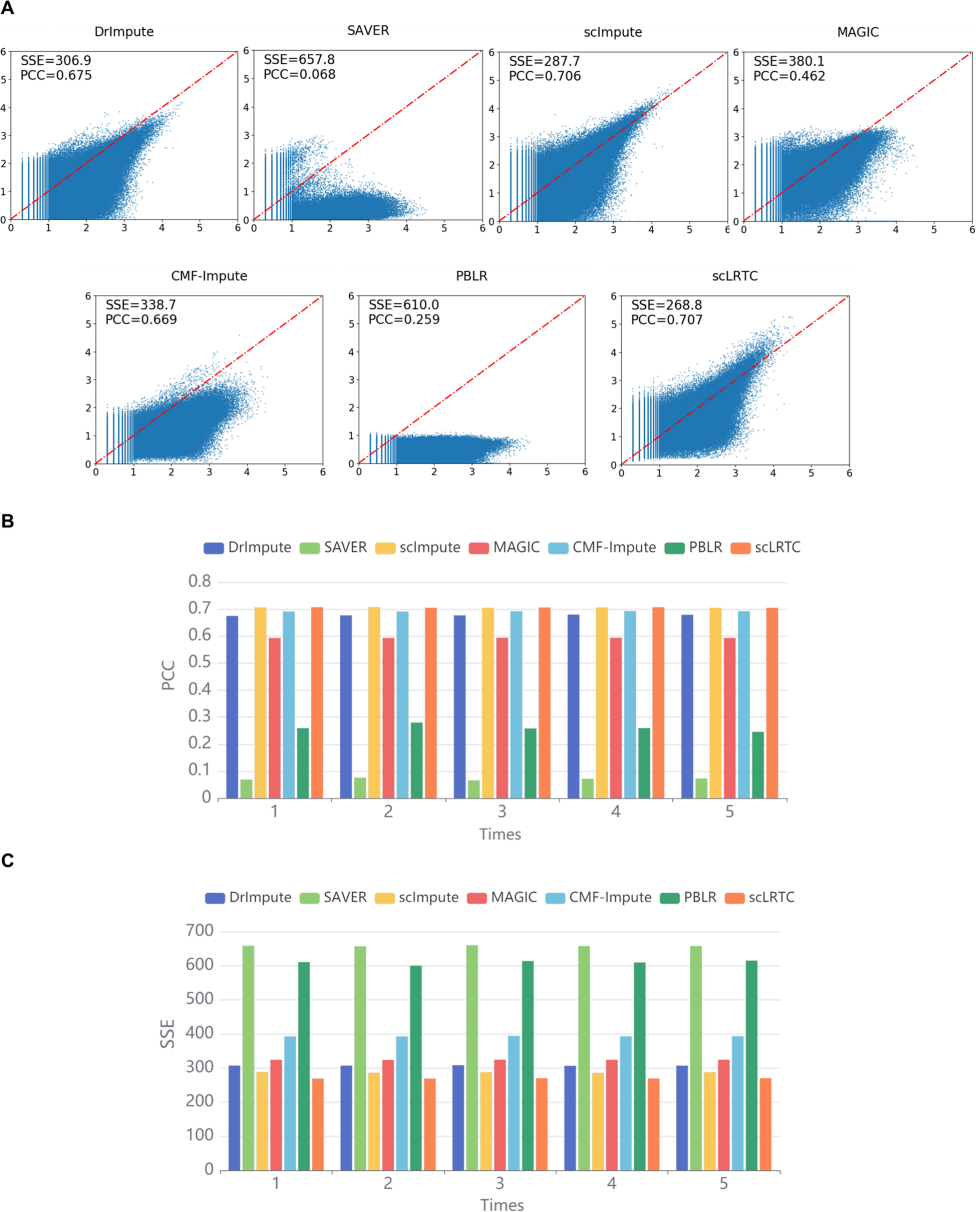
Imputation accuracy analysis and comparison of scLRTC and other methods in the real data masking. (A) Scatter plots of the masked imputation data and original data. The x-axis corresponds to the true value of the masked data point, and the y-axis represents the imputation value. The closer the points are to the red centerline, the higher the accuracy of imputaion. SSE and PCC are shown in the upper left corner (data is transformed by log_10_(*X* + 1)). (B) PCC values computed with 7 imputation methods in 5 repeated experiments. (C). SSE values computed with 7 imputation methods in 5 repeated experiments

The data masking evaluation was conducted on the real dataset and simulation datasets. First, 5% of non-zero entries were randomly selected from the Loh dataset, and these values were masked to zeros to generate a new gene expression matrix. Subsequently, seven imputation algorithms were applied for the new gene expression matrix and compared with unmasked data. The sum of squared errors (SSE) and Pearson correlation coefficient (PCC) between the imputation values and the true values were adopted to evaluate the effect of imputation. Figure 5A presents all the results of the imputation accuracy index of the masked data. The proposed method can recover the missing values with the lowest SSE of 268.8 and the highest PCC of 0.707 in all compared imputation algorithms. Note that the SAVER method persistently underestimates the values, especially among the highly expressed genes. Consistent experimental results were also mentioned in references [3, 15]. To prevent the influence attributed to randomness, we performed 5 masking repetitions for the above experiment. The results of 7 methods in 5 repeated experiments only slightly fluctuate (Fig. 5B and Fig. 5C), demonstrating that the randomness slightly impacts the mentioned results.

**Fig. 6:**
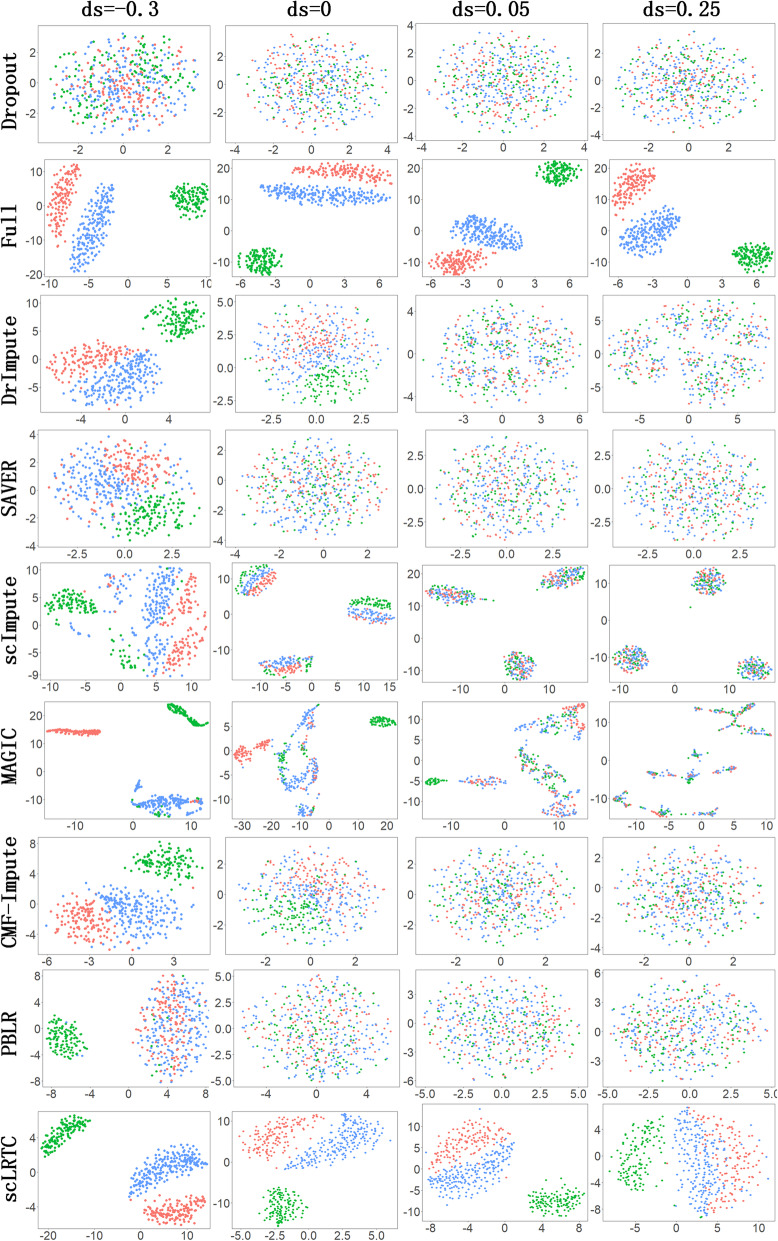
Visual distinguishability and comparison of scLRTC and other methods on simulation datasets with various dropout rates. We use t-SNE to visualize the cell gene expression matrix, and apply different algorithms for imputation. Each column represents a ds, which controls the ratio of zero

**Fig. 7:**
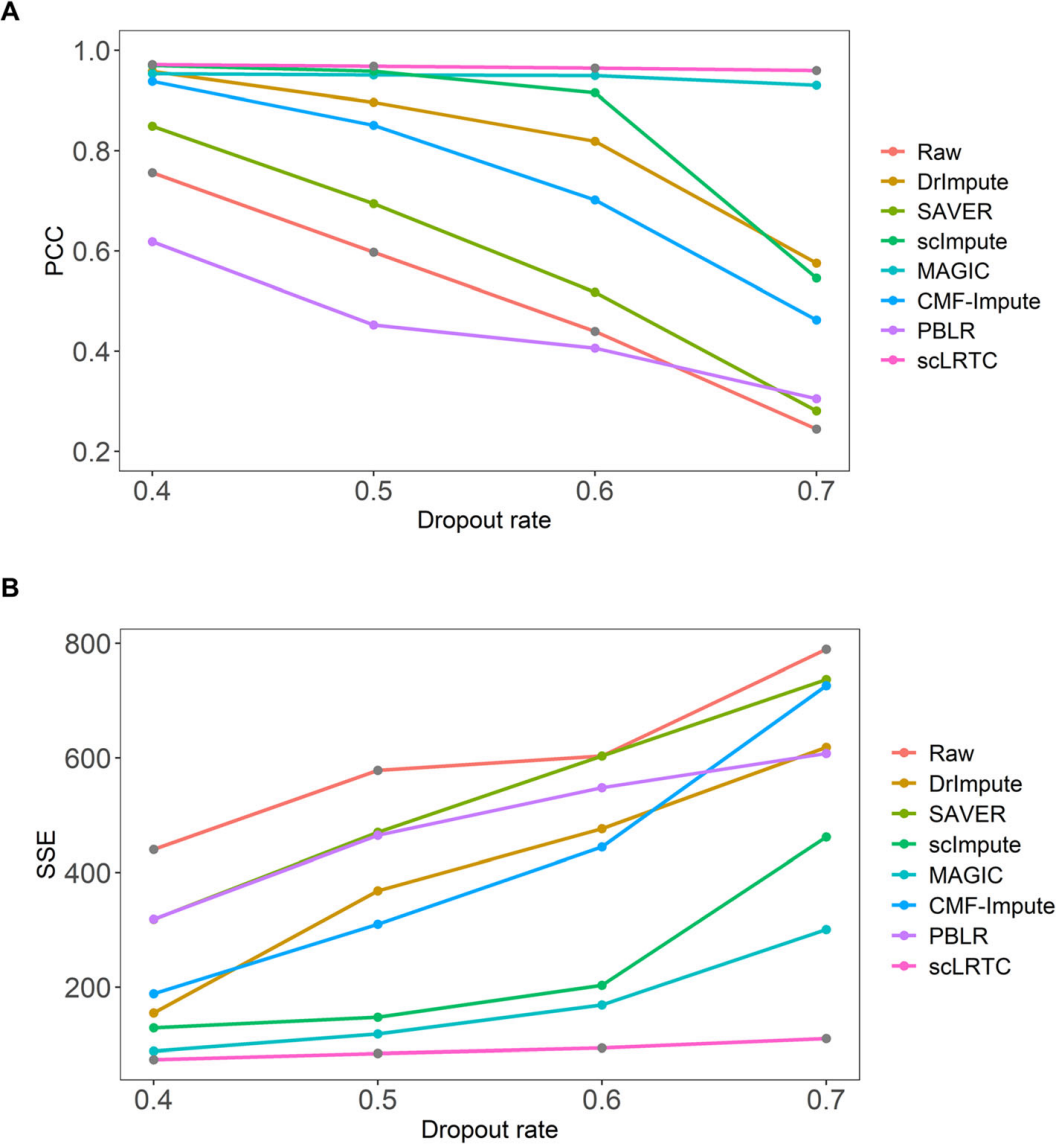
Imputation accuracy analysis and comparison of scLRTC and other methods under different dropout rates. (A) PCC values computed between the Full (without dropout) data and the raw data (with dropout) as well as imputed ones respectively. (B) SSE values computed between the Full data and the raw data as well as imputed ones respectively

Moreover, the performance of the proposed model was tested on single-cell simulation data that involves three cell populations. These data were generated using the Splatter package [29]. Splatter is an R bioconductor package for the reproducible and accurate simulation of scRNA-seq data. We referred to the parameters of simulation dataset provided by CMF-Impute [11] and increased the dropout rate in our experiment. Namely, 40, 50, 60, and 70% of the entries were randomly masked in the expression matrix, corresponding to a shape parameter of dropout logistic function (ds) equaling - 0.3, 0, 0.05, and 0.25 respectively. The masked entries were imputed with 7 methods and the imputed results are compared with the real values. Figure 6 shows the visualization results of t-SNE with dropout, unmasked raw data (Full), and 7 imputation methods (including DrImpute, scImpute, MAGIC, SAVER, CMF-Impute, PBLR and scLRTC) under different dropout rates. It can be seen that the proposed scLRTC is most consistent with the original data (Full) under the t-SNE visualization, demonstrating that the proposed imputation has a strong ability to restore real cell clusters. Furthermore, we performed the quantitative analysis on the simulation dataset. Figure 7 shows the SSE and PCC values under different dropout rates. With the increase in the dropout rate, the accuracy of all imputation methods is affected. However, the proposed scLRTC is suggested to exhibit the optimal performance among 7 methods.

### Evaluating imputation accuracy through correlation analysis and differential expression analysis

**Fig. 8:**
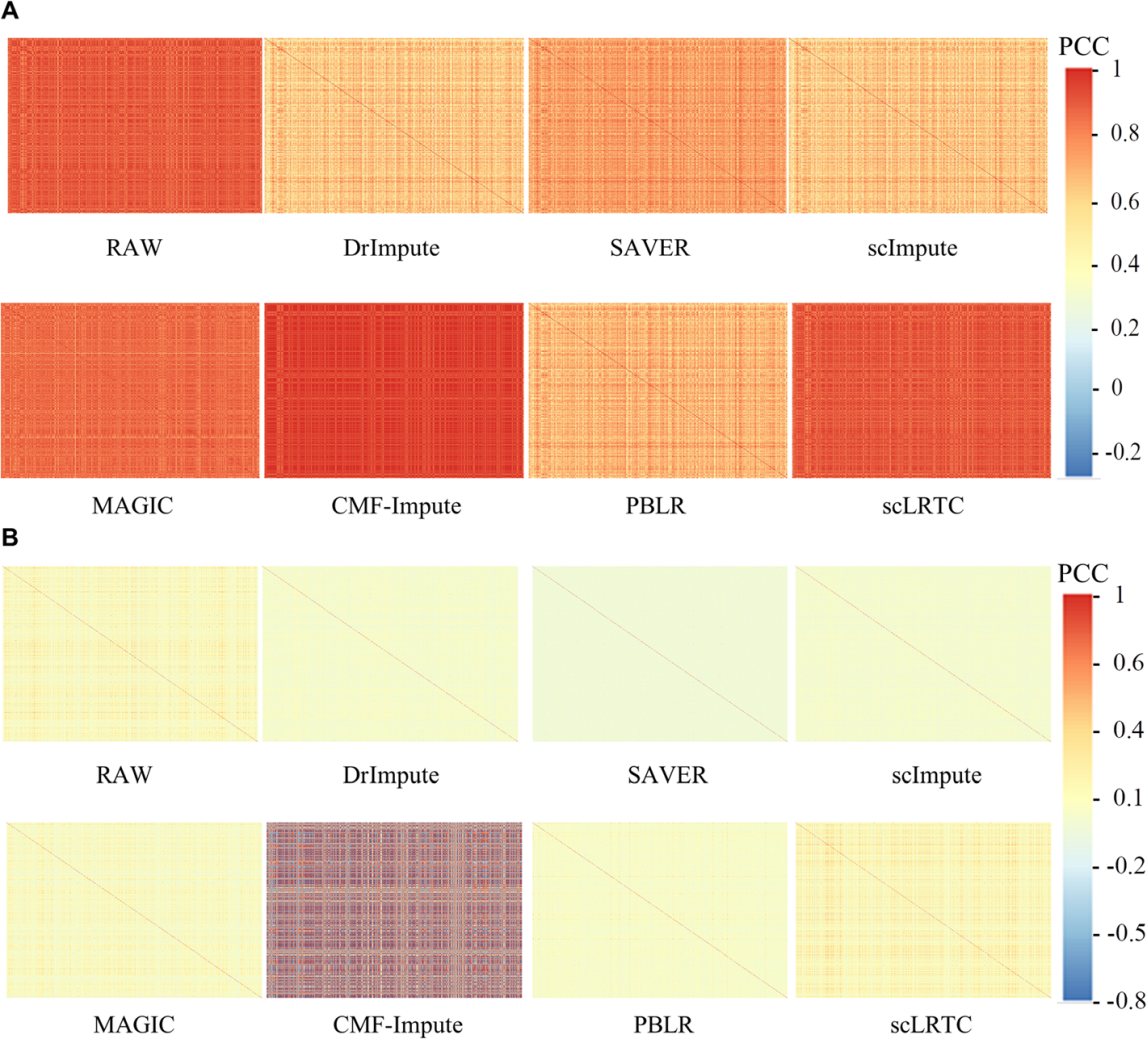
Correlation analysis and comparison of scLRTC and other methods. The more similar the heat map is to the raw heat map, the better the imputation effect. (**A)** Visualized heat map of cell-cell correlation matrix. **(B)** Visualized heat map of gene-gene correlation matrix

**Fig. 9:**
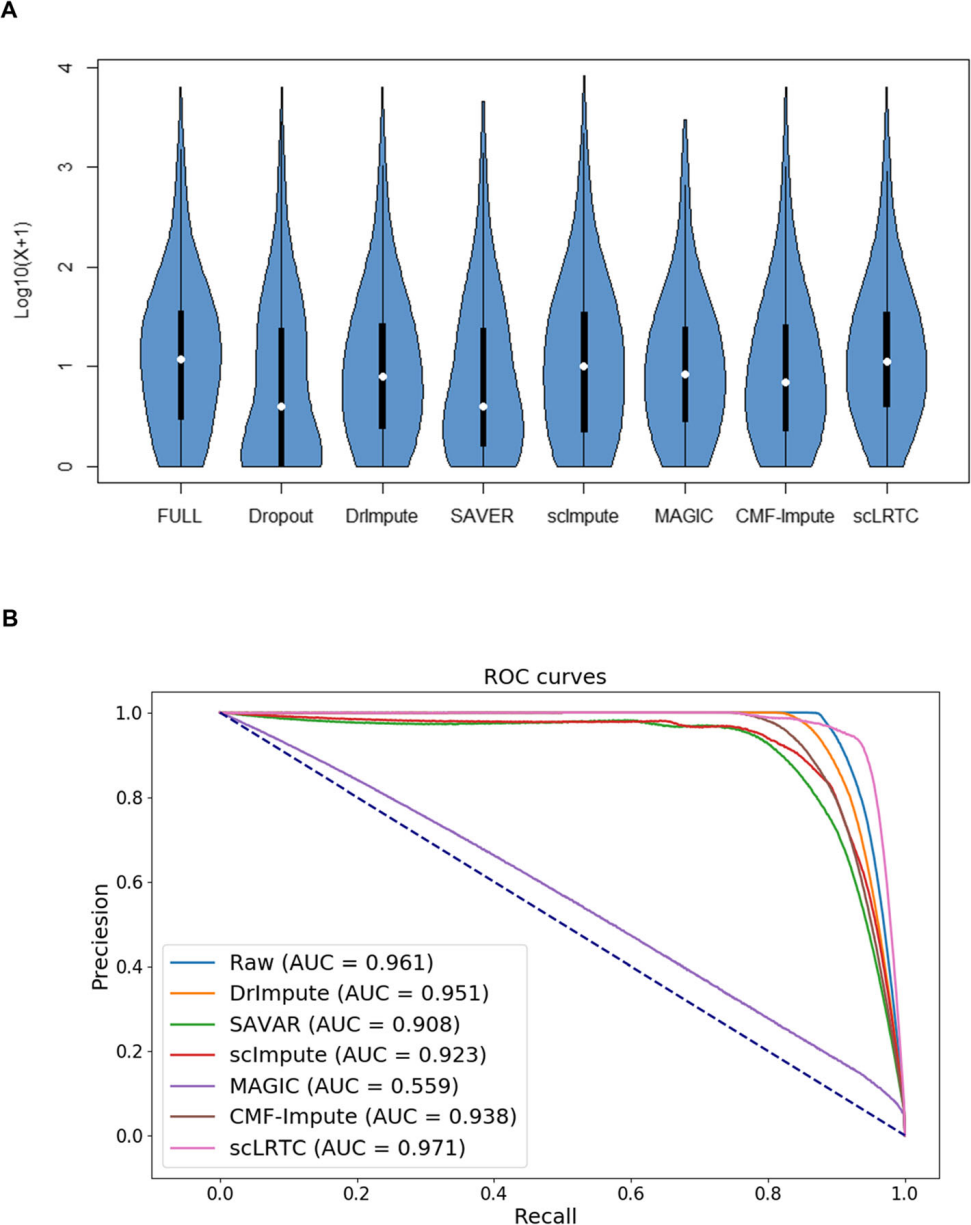
Violin chart of data expression distribution and accuracy measurements of DE genes by scLRTC and other methods. **(A)** Violin chart of data expression distribution after imputation when the dropout rate is 40% (data is transformed by log_10_(*X* + 1)). The more similar the shape of the violin is to FULL, the more effective the imputation effect. **(B)** ROC curves and AUC scores of DE genes with different imputation methods. AUC combines the recall rate and precision rate, and the value closer to 1 indicates a better imputation method. Here, the recall rate is defined as the number of true positives divided by the total number of samples that actually belong to the positive class, and the precision rate is the number of true positives divided by the total number of samples labelled as belonging to the positive class

The ability of the imputation method was evaluated to restore gene-gene and cell-cell relationships in complex tissues. The simulated data were employed with a dropout rate of 40% (ds = − 0.3) to calculate the gene-gene and cell-cell correlation matrix, and log_10_(*X* + 1) was set as the result after imputation. In the cell-to-cell correlation heat map (Fig. 8A), the color of MAGIC and the proposed scLRTC is the closest to the heat map of Full. For the heat map of gene-gene correlation (Fig. 8B), scImpute and the proposed scLRTC are the closest ones to the expression heat map of Full in color, while MAGIC deviates the most. And then the violin chart was used to display its expression distribution. We find the violin chart of scLRTC is the closest to the unmasked raw data (Full) in the appearance comparison (Fig. 9A), indicating that the position and the upper quartile comply with Full. It is suggested that the data by the scLRTC complement here achieves the most consistent distribution with that of Full. In summary, the proposed method can effectively restore the true gene-gene and cell-cell relationship.

In addition, it is considered that the imputation method should be capable of recovering true differential expressed (DE) genes and reducing the production of false positive genes. Since gold standard of DE genes has been rarely formulated in real datasets, 6 imputation methods (DrImpute, SAVER, scImpute, MAGIC, CMF-Impute and scLRTC) were compared for their capabilities to recover DE genes in the simulation data. The differential expression analysis was performed by using the MAST [39], and the true DE genes identified from the complete data were considered the reference. In terms of the respective method, the DE genes were extracted, which are considered significant by controlling *P*-value < 0.01 and comparing them with the true DE genes. Figure 9B presents the average ROC (Receiver Operating Characteristic) curves of different imputation methods by considering the indices of recall and precision. ScLRTC is found to achieve the highest score (AUC (Area Under the Curve) = 0.971) for detecting DE genes, demonstrating that scLRTC is valid to recover more DE genes and detect less false-positives genes.

### Evaluating imputation accuracy through cell trajectory inference

**Fig. 10:**
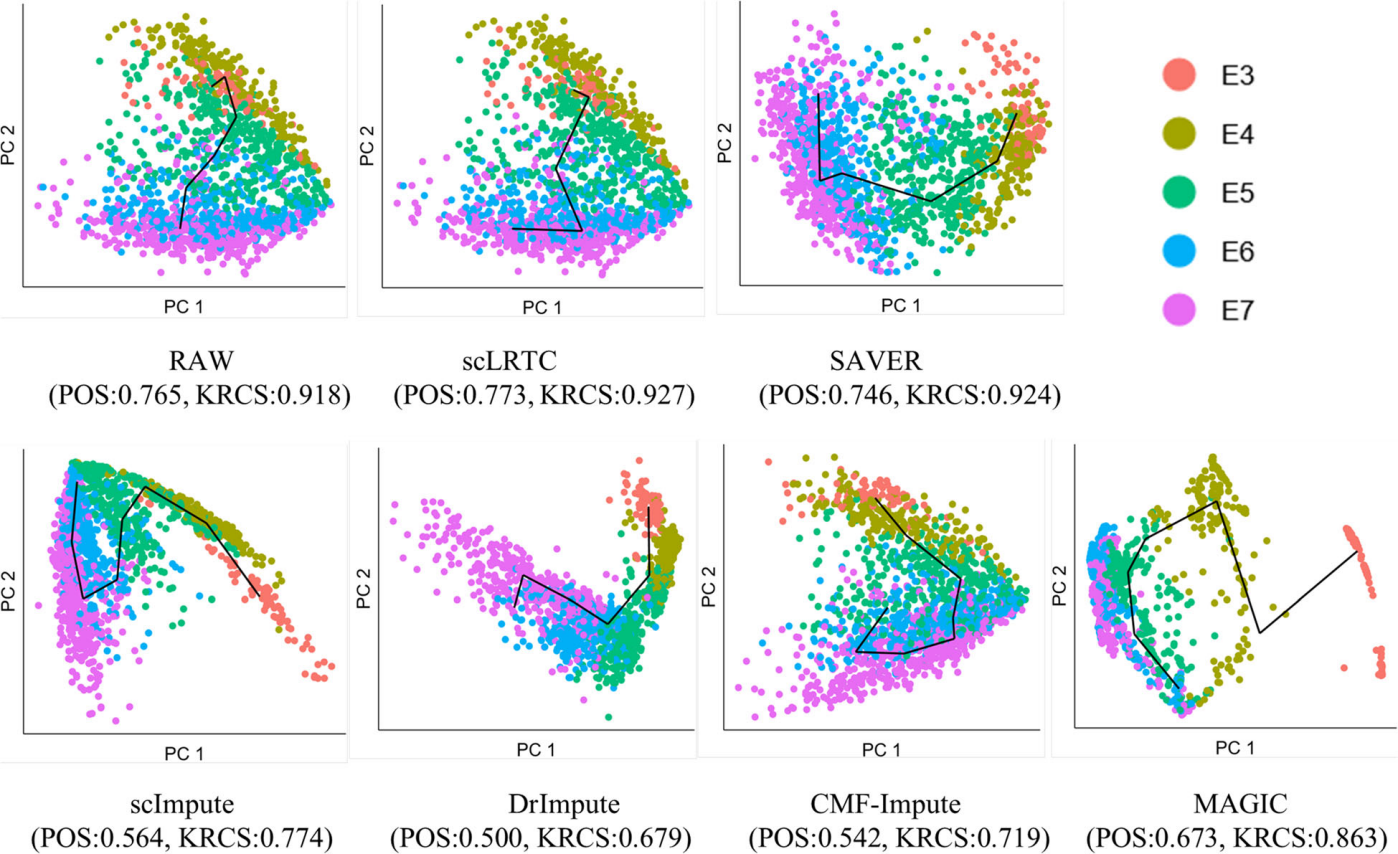
Cell trajectory inference analysis of scLRTC and other methods. Visualization of lineage reconstruction is implemented by TSCAN on the Petropoulos dataset. Lines represent the developmental trajectory of cells, and each type of cells (E3 to E7) represents a stage of cell development. Cells should distribute along the cell trajectory. The POS and Kendall’s rank correlation scores as the indicators to quantify this process are also provided

A common task of single cell RNA sequence analysis is to rebuild the lineage trajectory and infer the differentiation and progenitor status of single cells, which is a research hotspot over the past few years. Besides, a wide range of algorithms have been developed in this field. For instance, TSCAN [40] performed the differential expression and time series analysis on single-cell expression data, which classified individual cells according to the progress of biological processes. However, TSCAN did not perform dropout imputation for the data reprocessing. Thus, in this study, the scLRTC imputation was integrated into TSCAN, and its performance was compared in the pseudotime inference of the Petropoulos dataset. The Petropoulos data consists of the single cells from five stages of human preimplantation embryonic development from developmental day (E) 3 to day 7. Notably, though the cells at each time point may not be homogeneous, the time label can be exploited to represent an overall developmental trajectory. Accordingly, the known time label acts as the ground truth, and the performance of pseudotime inference is evaluated with TSCAN, as input by the raw data and the imputed data with 6 different methods (scLRTC, SAVER, scImpute, DrImpute, CMF-Impute and MAGIC). Furthermore, Pseudotime ordering score (POS) [40] and Kendall’s rank correlation score (KRCS) were used to measure the consistency of time label and pseudotime order derived from the data. The results are presented in Fig. 10.

It is therefore suggested that the proposed method has improved on both POS and KRCS indicators compared with the original data. For the SAVER method, it has an improvement on KRCS, whereas the POS score decreases. In terms of other methods, the pseudotime trajectory by DrImpute and scImpute starts at E3 and ends at E6, the pseudotime trajectory of CMF-Impute starts at E3 and ends at E5, and the trajectory reconstruction error is relatively large. The accuracy of MAGIC from E3 to E5 is relatively high, whereas at E6 and E7 stages, a big discrepancy is identified with the real label, which introduces errors.

## Discussion

Since single-cell RNA has a limited extraction efficiency, the occurrence of dropout events adversely affects the downstream analysis. However, the single-cell data imputation is not explicitly involved in the most used scRNA-seq tools for cell clustering, dimensionality reduction visualization, cell type recognition and lineage reconstruction, so it is of high research significance. In this paper, a novel low-rank tensor completion method (scLRTC) is proposed to impute the scRNA sequence data where dropout is present. ScLRTC, a data-driven method, fully considers the similarity and heterogeneity between cells. It builds a third-order tensor representation and employs a low-rank tensor completion model by adopting the ADMM algorithm to achieve the data imputation. This study also inputs the data with scLRTC imputation into SC3 clustering and carries out the clustering by first conducting t-SNE dimensionality reduction and then implementing K-means. Accordingly, it is reported that scLRTC is capable of increasing the clustering accuracy of real data at different dropout rates, as well as improving the quality of cell visualization. Moreover, by integrating the proposed scLRTC into TSCAN, we find it improves the accuracy of pedigree reconstruction and pseudotime inference.

Although we have verified that our scLRTC is superior to other popular methods on some real and simulation datasets, we cannot guarantee it is superior to all other methods on all datasets. We found our method is effective in imputing the scRNA-seq dataset with a high missing rate, mainly based on the following aspects. (1). Compared to ScImpute, PBLR and other clustering based methods, when the data missing rate is high, incorrect clustering result will affect the subsequent imputation process. Our scLRTC makes full use of the cell similarity to construct a low-rank tensor, which can reduce the impact of highly missing data on the imputation process. (2). For SAVER and other methods based on the statistical model, they normally impute the entire data under a given data distribution assumption. When the data distribution does not meet this assumption, the completion effect will be affected. But imputation by the low rank tensor of scRNA-seq data can avoid the influence of data distribution assumption. (3). For the scRNA-seq data, although the data itself has redundancy, the rank estimation of the original gene expression matrix is easy to be affected when the data has a high missing rate. Whereas, the rank estimation of the tensor constructed in our scLRTC can be tracked by the tensor trace norm, which can guarantee the final completion result.

In general, the proposed imputation method can be regarded as one powerful complement to current scRNA sequence data analysis. Our tensor based imputation algorithm can be further improved in the future work. For instance, because of the tensor model in scLRTC is relatively independent, we will develop the single-cell completion based on the parallel computing to improve the time complexity of scLRTC. Besides, we currently only use the similarity between cells to build a low-rank tensor. We can also consider the similarity between genes, and combine the similarity between cells and genes to build a higher-order tensor, and then complete the imputation under the tolerable computational complexity. In addition, we currently developed the scLRTC based on MATLAB mainly because there are the tensor related packages so that we can quickly develop our algorithm and verify it in the experiment analysis. In our future work, we will use R or Python to realize the scLRTC algorithm for providing the widespread use in the bioinformatics community.

## Conclusions

Imputation is an essential step in the use of scRNA-seq. In this work we introduced a novel low-rank tensor completion-based method, termed as scLRTC. Experiments on simulation data and real data sets showed scLRTC to be highly accurate in imputation.

## Methods

### Datasets

Nine scRNA-seq datasets (i.e., Pollen, Usoskin, Yan, Zeisel, Mouse, PBMC, Chen, Loh and Petropoulos) with different data sizes are used to test the validity of the proposed scLRTC in imputing dropout events. Besides, these datasets fall to three levels (i.e., gold, silver and copper) based on the supporting evidence of cell markers. To be specific, Pollen, Loh, Yan, Zeisel and Mouse datasets are defined as gold standard datasets, in which all cell markers are defined by complying with experimental conditions or cell lines. Usoskin, PBMC and Chen datasets are defined as the silver standard, with cell markers are calculated and assigned by drawing upon the authors’ knowledge of the underlying biology. Petropoulos is considered the copper standard since the cells involved are in the developmental stage (time labeled). Though single cell populations from different time points usually exhibit different expression patterns and biological characteristics, it remains infeasible to separate different populations at each time point based on time tags alone. Table 3 briefs these scRNA sequence datasets with sizes ranging from 90 (Yan) to 12,089 (Chen), and the number of cell clusters ranges from 4 (Usoskin) to 46 (Chen). Note that the first seven datasets are normally used for the cluster analysis. Furthermore, the first three datasets are from the low-throughput data sequencing platform, and the last four datasets originate from the high-throughput data sequencing platform. Loh is employed for the data masking evaluation, and Petropoulos is used for the trajectory reconstruction analysis.

**Table 3:** A summary of nine real scRNA-seq datasets used in our experiment

Dataset	Number of clusters	Number of cells	Number of genes	Standard
Pollen	11	301	23,730	Gold
Usoskin	4	622	25,334	Silver
Yan	7	90	20,214	Gold
Zeisel	9	3005	19,972	Gold
Mouse	16	2100	20,670	Gold
PBMC	8	4340	33,694	Silver
Chen	46	12,089	23,284	Silver
Loh	8	429	23,794	Gold
Petropoulos	5	1529	21,749	Copper

### Data preprocessing and normalization

In terms of a given scRNA-seq dataset, its gene expression matrix is recorded as *X*^*c*^. To reduce the effect of underexpressed genes, the gene expressed in less than or equal to 3 cells is removed [41]. To express the filtered matrix by *X*^*N*^, a matrix *X* is then made by taking the log_2_ transformation with a pseudo count 1

1 $$ {X}_{ij}={\log}_2\left({X}_{ij}^N+1\right);i=1,2,\dots, M,j=1,2,\dots, N $$

where *M* denotes the overall number of genes; *N* is the total number of cells. The pseudo-count is added to avoid infinite values in the parameter estimation in the subsequent data analysis. The logarithmic transformation has an advantage that it can prevent a small number of large observation values from being significantly affected in the data imputation.

### Tensor based model for scRNA-seq data imputation

Single-cell dropout events can be formulated as a missing value estimation problem. The core problem of missing value estimation refers to how to develop the relationship between known elements and unknown elements. The scRNA-seq data usually consist of single cells from different cell types, and single cells exhibiting the identical type have similar expression patterns. For this reason, it is assumed that the basic true expression of scRNA-seq data can be approximatively considered as a low-rank matrix. The low-rank matrix restoration essentially complies with the correlation between the rows and columns of a matrix, therefore creates a direct and effective imputation strategy.

A recent study suggested that taking full advantage of the presence of low-rank submatrix can improve the imputation performance compared to traditional low-rank matrix recovery methods [14, 15]. However, the low-rank submatrix constructed by clustering is easily influenced by the clustering effect, and the low-rank tensor can be constructed to capture more correlations of similar single cell compared to the low-rank submatrix form. Based on this motivation, the two-dimensional low-rank matrix is extended to the three-order low-rank tensor with the high correlation of scRNA-seq data. Besides, a novel low-rank tensor model is built for single-cell gene expression data, and the tensor trace norm [17] is employed to approximate the rank of the tensor, finally the missing data are rebuilt and the cell’s gene expression is restored.

### Tensor construction

We construct the tensor form of single-cell which fully considers the high correlation of scRNA-seq data. Specific to a given cell *X*_*i*_, the Pearson correlation coefficient between cells is first calculated and sorted in a descending order. Subsequently, the gene expression of cell *X*_*i*_ and its *K*-1 cells with the highest correlation are adopted to build a matrix *Mat*_*i*_ ∈ *ℝ*^*K* × *M*^, where *M* denotes the number of genes. Subsequently, the difference between cell *X*_*i*_ and other cells are measured in the matrix *Mat*_*i*_. To be specific, the Euclidean distance is calculated from all cells in *Mat*_*i*_ to *X*_*i*_ and sorted in an ascending order as *D*_*i*_ = (*d*_1*i*_, *d*_2*i*_, …, *d*_*Ki*_). Next, the direction (angle) similarity of cell gene expression is measured by calculating the cosine similarity from all cells in *Mat*_*i*_ to *X*_*i*_, and then it is recorded in an ascending order as *C*_*i*_ = (*c*_1*i*_, *c*_2*i*_, …, *c*_*Ki*_). Lastly, the similarity between cells is measured according to the absolute value of the difference of cell gene expressions, i.e., the Chebyshev distance from all cells in *Mat*_*i*_ to *X*_*i*_ is calculated and restored in an ascending order as *Q*_*i*_ = (*q*_1*i*_, *q*_2*i*_, …, *q*_*Ki*_).

**Fig. 11:**
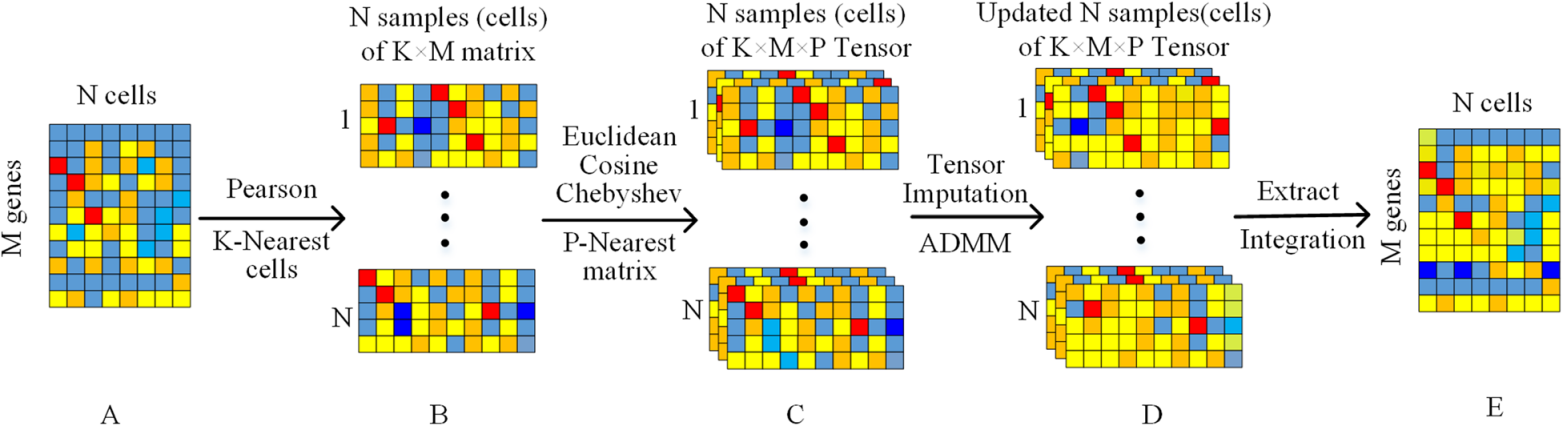
The whole framework of scLRTC. For the scRNA-seq dataset A, it uses the PCC and selects the closest *K* cells to construct *N K* × *M* low- rank matrices B. Then it applies the Euclidean, Cosine, and Chebyshev distances to select the closest *P* low-rank matrices to construct *N K* × *M* × *P* low-rank tensors C. Followingly, it uses the ADMM algorithm to impute the low-rank tensors C to obtain the updated tensors D. Finally, it extracts the cell vector from each low-rank tensor in D and integrates it to obtain the imputed scRNA-seq expression matrix E

Three distance vectors obtained from *X*_*i*_ are combined into a feature vector *Vec*_*i*_ = {*D*_*i*_, *C*_*i*_, *Q*_*i*_} with a size of 3*K* × 1. Likewise, the feature vector *Vec*_*j*_ can be obtained from the other cell *X*_*j*_. By calculating the distance between two feature vectors, the *P*-1 *Vec*_*j*_ closest to *Vec*_*i*_ is searched, and these matrices are merged to build a third-order tensor $\mathbf{\mathcal{Y}}\in {\mathbb{R}}^{K\times M\times P}$ for the cell *X*_*i*_ (as shown in Fig. 11).

Our method fully exploits the similarity between cells to construct a low-rank tensor, which can avoid the imputation influence by the high missing rate of scRNA-seq data in the completion process. On the other hand, the tensor trace norm is used to track the rank of tensor and solved by the ADMM algorithm, which can guarantee the imputation results more accurate and robust compared with other heuristic tensor completion methods (Tucker, Parafac and SVD).

### Tensor fold and unfold

During the tensor analysis, it is convenient to unfold a tensor into a matrix. The “unfold” operation along the *k* th mode on a tensor $\mathbf{\mathcal{Y}}$ is defined as

2 $$ {unfold}_k\left(\mathbf{\mathcal{Y}}\right)={\mathbf{\mathcal{Y}}}_{(k)}\in {\mathbb{R}}^{I_k\times \left({I}_1\dots {I}_{k-1}{I}_{k+1}\dots {I}_n\right)} $$

The opposite operation “fold” is defined as

3 $$ {fold}_k\left({\mathbf{\mathcal{Y}}}_{(k)}\right)=\mathbf{\mathcal{Y}} $$

### Tucker decomposition and denoising

Tensor Tucker decomposition is recognized as a form of high-order principal component analysis. The HOSVD method [42] is available for decomposing a third-order tensor $\mathbf{\mathcal{Y}}\in {\mathbb{R}}^{I\times J\times K}$ by

4 $$ \mathbf{\mathcal{Y}}\approx \mathbf{\mathcal{G}}{\times}_1A{\times}_2B{\times}_3C $$

where *A* ∈ *ℝ*^*I* × *P*^, *B* ∈ *ℝ*^*J* × *Q*^ and *C* ∈ *ℝ*^*K* × *R*^ denote factor matrices, which can be considered the main components of the corresponding mode. The tensor $\mathbf{\mathcal{G}}\in {\mathbb{R}}^{P\times Q\times R}$ refers to the core tensor, representing the level of interaction between different components.

Next, a hard threshold function is set for factor matrices *A*, *B*, *C* to eliminate the effect of some low value components after the Tucker decomposition. Subsequently, the third-order tensor $\hat{\mathbf{\mathcal{Y}}}$ is restored by updating $\hat{\mathbf{\mathcal{G}}}$, i.e., the convergence of the current tensor data is ensured by the iterative computation, and the denoising effect of some mutation elements is achieved in the tensor form of scRNA-seq data.

### Tensor trace norm

The trace norm of a tensor is defined as [17].

5 $$ {\left\Vert \mathbf{\mathcal{Y}}\right\Vert}_{\ast }={\sum}_{i=1}^n{\alpha}_i{\left\Vert {\mathbf{\mathcal{Y}}}_{(i)}\right\Vert}_{\ast } $$

where ${\alpha}_i\ge 0,{\sum}_{i=1}^n{\alpha}_i=1$, ${\mathbf{\mathcal{Y}}}_{(i)}$ expresses the matrix along the *i* th mode. In fact, the trace norm of a tensor refers to a convex combination of the trace norms of all matrices expanded along the respective mode. Notably, when *n* is equal to 2 (the matrix case), the definition of the tensor’s trace norm complies with the matrix case.

### Tensor completion model

By using *M*_*i*_ to replace $\mathbf{\mathcal{Y}}$, the tensor completion model is expressed as

6 $$ {\displaystyle \begin{array}{c}{\mathit{\min}}_{y,{M}_1,\dots, {M}_n}{\sum}_{i=1}^n{\alpha}_i{\left\Vert {M}_{i(i)}\right\Vert}_{\ast}\\ {}s.t.{Y}_{\varOmega }={T}_{\varOmega}\\ {}Y={M}_i,i=1,\dots, n.\end{array}} $$

where *α*_*i*_ denotes the coefficient; *M*_*i*(*i*)_ represents the unfold matrix of the tensor along the ith mode; $\mathbf{\mathcal{T}}$ is the known tensor; $\mathbf{\mathcal{Y}}$ expresses the reconstructed tensor; *Ω* in ${\mathbf{\mathcal{T}}}_{\varOmega }$ is the index of non-zero observation value.

### Tensor model solution

The mentioned model can be solved by adopting the alternating direction method of multipliers (ADMM) algorithm. The augmented Lagrangian function is defined as

$$ {L}_{\rho}\left(\mathbf{\mathcal{Y}},{M}_1,\dots, {M}_n,{y}_1,\dots, {y}_n\right)=\sum \limits_{i=1}^n{\alpha}_i{\left\Vert {M}_{i(i)}\right\Vert}_{\ast }+\left\langle \mathbf{\mathcal{Y}}-{M}_i,{y}_i\right\rangle + $$

7 $$ \frac{\rho }{2}{\left\Vert {M}_i-\mathbf{\mathcal{Y}}\right\Vert}_F^2 $$

where < ∙ , ∙ > denotes the inner product; ${\left\Vert \bullet \right\Vert}_F^2$ represents the F-norm, i.e., the root of the square sum of all elements; *y*_*i*_ is the Lagrange multiplier; *ρ* expresses the penalty parameter.

According to the framework of ADMM, ${M}_i,\mathbf{\mathcal{Y}},{y}_i$ can be iteratively updated as

I $$ \left\{{M}_1^{k+1},\dots, {M}_n^{k+1}\right\}={argmin}_{M_1,\dots, {M}_n}{L}_{\rho}\left({\mathbf{\mathcal{Y}}}^k,{M}_1,\dots, {M}_n,{y}_1^k,\dots, {y}_n^k\right) $$

II $$ {\mathbf{\mathcal{Y}}}^{k+1}={argmin}_{\mathbf{\mathcal{Y}}}\ {L}_{\rho}\left(\mathbf{\mathcal{Y}},{M}_1^{k+1},\dots, {M}_n^{k+1},{y}_1^k,\dots, {y}_n^k\right) $$

III $$ {y}_i^{k+1}={y}_i^k-\rho \left({M}_i^{k+1}-{\mathbf{\mathcal{Y}}}^{k+1}\right) $$

From the augmented Lagrangian function in 1), ${\mathbf{\mathcal{Y}}}_{(i)}^k,{y}_i^k$ is fixed and minimized to yield

$$ {M}_{i(i)}^{k+1}=\mathit{\arg}\underset{M_{i(i)}}{\mathit{\min}}{\alpha}_i{\left\Vert {M}_{i(i)}\right\Vert}_{\ast }+\frac{\rho }{2}\left\langle {M}_{i(i)},{M}_{i(i)}\right\rangle -\rho \left\langle {M}_{i(i)},{\mathbf{\mathcal{Y}}}_{(i)}^k+\frac{1}{\rho }{y}_{i(i)}^k\right\rangle $$

$$ =\mathit{\arg}\underset{M_{i(i)}}{\mathit{\min}}{\alpha}_i{\left\Vert {M}_{i(i)}\right\Vert}_{\ast }+\frac{\rho }{2}{\left\Vert {M}_{i(i)}-{\mathbf{\mathcal{Y}}}_{(i)}^k-\frac{1}{\rho }{y}_{i(i)}^k\right\Vert}_F^2 $$

8 $$ -\left\langle {\mathbf{\mathcal{Y}}}_{(i)}^k+\frac{1}{\rho }{y}_i^k,{\mathbf{\mathcal{Y}}}_{(i)}^k+\frac{1}{\rho }{y}_{i(i)}^k\right\rangle $$

Thus, the optimal solution of ${M}_{i(i)}^{k+1}$ is

9 $$ {M}_{i(i)}^{k+1}=\mathit{\arg}\underset{M_{i(i)}}{\mathit{\min}}{\alpha}_i{\left\Vert {M}_{i(i)}\right\Vert}_{\ast }+\frac{\rho }{2}{\left\Vert {M}_{i(i)}-\left({\mathbf{\mathcal{Y}}}_{(i)}^k+\frac{1}{\rho }{y}_{i(i)}^k\right)\right\Vert}_F^2 $$

The above Eq. (9) is proven to generate a closed-form in recent references [43, 44], so it can be solved by calculating the singular value thresholding operator *D*_*τ*_(∙). In terms of any matrix *X*, the singular value decomposition (SVD) is performed to obtain *X* = *UΣV*^*T*^, where *U*, *V* are orthogonal singular vectors, and *Σ* ∈ R^*r* × *r*^ comprises singular values *σ*_1_, …, *σ*_*r*_, *r* =  *min* {*m*, *n*}. The singular value thresholding operator can be defined as *D*_*τ*_(*X*) = *UΣ*_*τ*_*V*^*T*^, where Σ_τ_= *diag*(*max*(*σ*_*i*_ − *τ*, 0)). Thus, it yields

10 $$ {M}_{i(i)}={D}_{\frac{\alpha_i}{\rho }}\left({\mathbf{\mathcal{Y}}}_{(i)}+\frac{1}{\rho }{y}_{i(i)}\right) $$

By folding *M*_*i*(*i*)_ to get

11 $$ {M}_i={fold}_i\left({M}_{i(i)}\right) $$

From the augmented Lagrangian function in 2), it can be minimized by fixing ${M}_i^k$ and ${y}_i^k$, and the optimal solution is obtained as

$$ {\displaystyle \begin{array}{c}{\mathbf{\mathcal{Y}}}^{k+1}=\mathit{\arg}\underset{\mathbf{\mathcal{Y}}}{\mathit{\min}}\sum \limits_{i=1}^n\left\langle \mathbf{\mathcal{Y}},{y}_i^k\right\rangle +\frac{\rho }{2}\left\langle \mathbf{\mathcal{Y}}-{M}_i^{k+1},\mathbf{\mathcal{Y}}-{M}_i^{k+1}\right\rangle \\ {}\ \end{array}} $$

12 $$ =\mathit{\arg}\underset{\mathbf{\mathcal{Y}}}{\mathit{\min}}\sum \limits_{i=1}^n\frac{\rho }{2}\left\langle \mathbf{\mathcal{Y}},\mathbf{\mathcal{Y}}\right\rangle +\left\langle \mathbf{\mathcal{Y}},{\rho M}_i^{k+1}-{y}_i^k\right\rangle $$

Take the derivative of (12) with respect to $\mathbf{\mathcal{Y}}$ and set it equal to 0 to yield

13 $$ n\rho \mathbf{\mathcal{Y}}-\left(\sum \limits_{i=1}^n{\rho M}_i^{k+1}-{y}_i^k\right)=0 $$

So

14 $$ {\mathbf{\mathcal{Y}}}^{k+1}=\frac{1}{n}\left({\sum}_{i=1}^n{M}_i^{k+1}-\frac{1}{\rho }{y}_i\right) $$

For $\mathbf{\mathcal{Y}}={\mathbf{\mathcal{Y}}}_{\overline{\varOmega}}+{\mathbf{\mathcal{Y}}}_{\varOmega }$, ${\mathbf{\mathcal{Y}}}_{\varOmega }={\mathcal{T}}_{\varOmega }$ is known, so only ${\mathbf{\mathcal{Y}}}_{\overline{\Omega}}$ is updated

15 $$ {\mathbf{\mathcal{Y}}}_{\overline{\varOmega}}^{k+1}=\frac{1}{n}{\left({\sum}_{i=1}^n{M}_i^{k+1}-\frac{1}{\rho }{y}_i^k\right)}_{\overline{\varOmega}} $$

After the tensor completion reconstruction, the corresponding cell expression *X*_*i*_(*i* = 1, 2, …, *N*) is selected from the respective tensor model, and the gene information of each cell is restored, and a matrix *X*^∗^ representing the complete scRNA-seq data is lastly formed. Since the gene expression of the cell is non-negative, the matrix *X*^*P*^ is defined after the imputation

16 $$ {X}^P=\left\{\begin{array}{c}{X}_{ij}^P=0\  if\left({X}_{ij}^{\ast }<0\right)\\ {}{X}_{ij}^P={X}_{ij}^{\ast }\  others\ \end{array}\ \right. $$

In brief, the entire scLRTC algorithm process is expressed as.

**Table:** 

**Input** : *X*, *ρ*, *α and K* $\mathbf{Output}:\hat{X}$ (*X* after imputing) 1. **for** *m* = 1 *to N* 2. Construction of low rank tensor *Τ*_Ω_ of *X*_*m*_ $3:\kern1.25em \mathrm{Set}\ {\mathbf{\mathcal{Y}}}_{\Omega}={T}_{\Omega}\ \mathrm{and}\ {\mathbf{\mathcal{Y}}}_{\overline{\Omega}}=0,\mathbf{\mathcal{Y}}={M}_i$ 4 : **for** *k* = 0 *to K* **do** 5 : **for** *i* = 1 *to n* **do** $6:\kern5em {M}_{i(i)}={\boldsymbol{D}}_{\frac{\alpha_i}{\rho }}\left({\mathbf{\mathcal{Y}}}_{(i)}+\frac{1}{\rho }{y}_{i(i)}\right)$ 7 : *M*_*i*_ = *fold*_*i*_[*M*_*i*(*i*)_] 8 : **end for** $9:\kern2.75em {\mathbf{\mathcal{Y}}}_{\overline{\varOmega}}=\frac{1}{n}{\left({\sum}_{i=1}^n{M}_i-\frac{1}{\rho }{y}_i\right)}_{\overline{\varOmega}}$ $10:\kern2.25em {y}_i={y}_i-\rho \left({M}_i-\mathbf{\mathcal{Y}}\right)$ 11 : **end for** 12:extract ${\hat{X}}_m$ from $\mathbf{\mathcal{Y}}$ 13 : **end for** 14: ${X}^{\ast }=\left(\ {\hat{X}}_1,{\hat{X}}_2,\dots, {\hat{X}}_m\right)$ and use Eq. (16) to remove negative values	

### Evaluation measures

To objectively evaluate the effectiveness of the proposed low-rank tensor completion method for single-cell RNA-seq data, the reconstructed data are used for the cell clustering, and two clustering indicators with the normalized mutual information (NMI) and the adjusted rand index (ARI) are adopted to quantify the consistency between inferred and predefined cell clusters in the respective scRNA-seq data. Subsequently, the silhouette coefficient (SC) is adopted to assess the visual effect of cell dimensionality reduction. Lastly, Pseudotemporal ordering score (POS) Tand KRCS are used to evaluate the accuracy of cell trajectory analysis and imputation.

Denote that *U* = {*μ*_1_, *μ*_2_, …, *μ*_*P*_} is adopted to represent the true partition of *P* classes, *V* = {*v*_1_, v_2_, …, *v*_*K*_} is used to denote the partition given by clustering results, *n*_*i*_ and *n*_*j*_ are represented as the number of the class *μ*_*i*_ and cluster *v*_*j*_, respectively, and *n*_*ij*_ is expressed as the number of observations in both class *μ*_*i*_ and cluster *v*_*j*_.

ARI is then formulated as:

17 $$ \frac{\sum_{i=1}^P{\sum}_{j=1}^K\left(\genfrac{}{}{0pt}{}{n_{ij}}{2}\right)-\left[{\sum}_{i=1}^P\left(\genfrac{}{}{0pt}{}{n_{i\bullet }}{2}\right){\sum}_{j=1}^K\left(\genfrac{}{}{0pt}{}{n_{\bullet j}}{2}\right)\right]/\left(\genfrac{}{}{0pt}{}{n}{2}\right)}{\frac{1}{2}\left[{\sum}_{i=1}^P\left(\genfrac{}{}{0pt}{}{n_{i\bullet }}{2}\right){\sum}_{j=1}^K\left(\genfrac{}{}{0pt}{}{n_{\bullet j}}{2}\right)\right]-\left[{\sum}_{i=1}^P\left(\genfrac{}{}{0pt}{}{n_{i\bullet }}{2}\right){\sum}_{j=1}^K\left(\genfrac{}{}{0pt}{}{n_{\bullet j}}{2}\right)\right]/\left(\genfrac{}{}{0pt}{}{n}{2}\right)} $$

where $$ n=\sum \limits_{i=1}^P{n}_{i\bullet }=\sum \limits_{j=1}^K{n}_{\bullet j}. $$

NMI is expressed as

18 $$ NMI=\frac{2I\left(U,V\right)}{H(U)+H(V)} $$

where *I*(*U*, *V*) expresses the amount of mutual information between *U* and *V*

19 $$ I\left(U,V\right)={\sum}_{i=1}^P{\sum}_{j=1}^K\frac{\left|{u}_i\cap {\upsilon}_j\right|}{N}\log \frac{N\left|{u}_i\cap {\upsilon}_j\right|}{\left|{u}_i\right|\times \left|{\upsilon}_j\right|} $$

*H*(*U*) and *H*(*V*) are the entropies of partitions *U* and *V*

20 $$ H(U)=-{\sum}_{i=1}^P\frac{u_i}{N}\mathit{\log}\frac{u_i}{N},H(V)=-{\sum}_{i=1}^K\frac{v_i}{N}\mathit{\log}\frac{v_i}{N} $$

where *N* is the total number of cells.

SSE is written as

21 $$ \mathrm{sqrt}\left({\sum}_{i=1}^n{\sum}_{i=1}^n{\left({X}_{ij}-{P}_{ij}\right)}^2\right) $$

where *X*_*ij*_ denotes the true gene expression; *P*_*ij*_ represents the predicted gene expression.

SC is expressed as

22 $$ SC=\mathrm{average}\left({\sum}_{i=1}^N\frac{b(i)-a(i)}{\max \left\{a(i),b(i)\right\}}\right) $$

where *i* denotes the *i* th cell, *a*(*i*) = *average* (*i* to all other cells in the cluster to which it belongs), *b*(*i*) = *min* (the average distance from *i* to all cells in the other cluster).

## Data Availability

There are no new data associated with this article. Published datasets used in this study: Pollen et al.’s dataset ([20], P041736) of human tissues cells is available at https://www.ncbi.nlm.nih.gov/sra?term=SRP041736. Usoskin et al.’s dataset ([21], GSE59739) of mouse lumbar dorsal root ganglion cells is available at https://www.ncbi.nlm.nih.gov/geo/query/acc.cgi?acc=GSE59739. Yan et al.’s dataset ([22], GSE36552) of the human embryos cells is available at https://www.ncbi.nlm.nih.gov/geo/query/acc.cgi?acc=GSE36552. Zeisel et al.’s dataset ([23]; GSE60361) of the mouse cortex and hippocampus cells is available at https://www.ncbi.nlm.nih.gov/geo/query/acc.cgi?acc=GSE60361. The mouse bladder cell dataset [24] originates from the mouse cell atlas project is available at https://figshare.com/s/865e694ad06d5857db4b. From the raw count matrix, 2100 cells are selected from the bladder tissue. PBMC dataset [25] is downloaded from the 10X genomics website (https://support.10xgenomics.com/single-cell-gene-expression/datasets/2.1.0/pbmc4k). Chen et al.’s dataset ([26], GSE87544) of adult mouse hypothalamus cells is available at https://www.ncbi.nlm.nih.gov/geo/query/acc.cgi?acc=GSE87544. Loh et al.’s dataset ([27], SRP 073808) of human embryonic stem cells is available at https://www.ncbi.nlm.nih.gov/sra/?term=SRP073808. Petropoulos et al.’s dataset ([28], E-MTAB-3929) of human preimplantation embryos cells is available at https://www.ebi.ac.uk/arrayexpress/experiments/E-MTAB-3929. All the source codes and supplementary data are available at https://github.com/jianghuaijie/scLRTC.

## Abbreviations

scRNA-seq: Single-cell RNA-sequencing
PCC: Pearson correlation coefficient
SSE: sum of squares of error
ARI: Adjusted Rand Index
NMI: Normalized Mutual Information
SC: silhouette coefficient
POS: Pseudotemporal ordering score
KRCS: Kendall’s rank correlation score
ADMM: alternating direction method of multipliers
